# Peters plus syndrome and Chorioretinal findings associated with *B3GLCT* gene mutation - a case report

**DOI:** 10.1186/s12886-020-01380-6

**Published:** 2020-03-23

**Authors:** Ye Elaine Wang, Dhariana Acon Ramirez, Ta Chen Chang, Audina Berrocal

**Affiliations:** 1 Bascom Palmer Eye Institute, University of Miami, 900 NW 17th St, Miami, FL 33136 USA; 2 Harvard Eye Associates, Laguna Hills, CA 92653 USA

**Keywords:** Peters plus syndrome, Peters anomaly, B3GLCT mutation, Anterior segment dysgenesis, Atrophic chorioretinal lesions

## Abstract

**Background:**

Peters plus syndrome (PPS) is a combination of congenital Peters anomaly and systemic abnormalities. It is inherited most commonly in an autosomal recessive pattern with homozygous *B3GLCT* mutations. Ocular findings consist predominantly anterior segment abnormalities without posterior segment involvement.

**Case presentation:**

In this presentation, we report a case of PPS with homozygous pathogenic variant in *B3GLCT* who presented with classic anterior segment findings, systemic abnormalities, as well as atypical bilateral chorioretinal atrophy. The chorioretinal findings were characterized with spectral-domain optical coherence tomography.

**Conclusions:**

Our report expands the phenotypic descriptions of PPS by characterizing posterior segment findings.

## Background

Peters plus syndrome (PPS, also known as Krause-Kivlin or Krause-van Schooneveld-Kivlin syndrome) is characterized by eye abnormalities, characteristic facial features, cleft lip/palate, short limbs with brachydactyly, and developmental delay with intellectual disability [1].

PPS is usually inherited in an autosomal recessive pattern due to biallelic mutations in *B3GLCT* (also known as *B3GALTL*), which is associated with an increased risk for miscarriages and stillbirth of affected fetuses [2–4]. Systemic abnormalities may include congenital heart defects, genitourinary anomalies, structural brain malformations, congenital hypothyroidism, and conductive hearing loss [1, 3, 4].

Eye abnormalities in PPS predominantly involve the anterior segment, including corneal clouding, posterior cornea thinning, and iridocorneal adhesions. Based on the anatomic pathology, Peters anomaly may be classified as type I (without keratolenticular adhesion), or type II (with lens abnormalities) [4]. Other ophthalmological defects include iris coloboma and retrolental cyst, although no optical tomographic characterization of chorioretinal findings has been described [1, 5]. In this report, we characterize the chorioretinal findings in a patient with PPS with homozygous pathogenic *B3GLCT* mutation.

## Case presentation

The patient is a 22-month-old Caucasian boy, born at 36 weeks by spontaneous vaginal delivery with normal prenatal care. He had no family history of glaucoma or anterior segment dysgenesis. At birth, he was noted to have bilateral cloudy corneas with no epiphora nor photophobia. He was started on presumptive treatment of topical dorzolamide 2% and hypertonic sodium chloride ophthalmic solution 5% prior to his presentation at the Bascom Palmer Eye Institute. A bedside eye exam revealed a “corneal opacity centrally sparing the periphery” and he was diagnosed with possible congenital glaucoma associated with Peters anomaly. Extraocular findings included short extremities and brachydactyly, cleft palate, characteristic facial features, developmental delay, and pulmonary stenosis (Table 1).

**Table 1:** Summary of ocular and systemic findings in patients with Peters Anomaly Type 1 and 2, Peters Plus Syndrome, and Our Patient

	Peters Anomaly Type 1	Peters Anomaly Type 2	Peters Plus Syndrome With *B3GLCT* gene mutations ^a^	Our Patient
**Ocular Findings**				
Central corneal clouding	+	+	+	+
Iris-corneal adhesion	+	+	+	+
Lens-corneal adhesion	–	+	+	–
Cataract	possible	possible	possible	+
Glaucoma	possible	possible	possible	possible
Chorioretinal lesions	–	–	rare^b^	+
**Systemic Findings**				
Short limbs with broad distal extremities	–	–	+	+
Cleft Palate	–	–	+	+
Characteristic facial features	–	–	+	+
Developmental delay/Intellectual disabilities	–	–	+	+
Heart Defects	–	–	possible	+^c^
Genitourinary	–	–	possible	–
Hearing	–	–	possible	–
Brain abnormalities	–	–	possible	–

+Present; − Absent

^a^ All possible mutation including homozygous and compound heterozygous variants. Not necessairly the same point mutation as our patients

^b^Two independent reports in literature with no characterization of lesions

^c^ Pulmonary stenosis

An exam under anesthesia (EUA) was performed the same day. Intraocular pressures (IOP) were 32 mmHg and 27 mmHg in the right eye (OD) and left eye (OS), respectively (Tono-Pen XL, Reichert Inc. Depew, NY). Bright red reflexes were noted prior to dilation in both eyes (OU). Other ocular abnormalities included iridocorneal adhesion superiorly OS and posterior capsular cataract OU. Given the absence of limbal enlargement, normal axial lengths, and an intact red reflex prior to dilation, glaucoma surgery was deferred. Phenylephrine 2.5% OU was added for optical dilation and visual rehabilitation, and topical dorzolamide 2% was continued.

Four months later, a second EUA was performed, IOPs were 30 mmHg OD and 35 mmHg OS by Tono-pen. The corneal opacities were noted to have enlarged centrally, though the clear peripheral cornea still maintained intact red reflexes. The irides were noted to have formed whispy attachments to the corneas in both eyes. The view of the lens and posterior structures at this point were suboptimal. A diagnosis of Peters anomaly type 1 was made. Genetic testing subsequently identified a homozygous pathogenic variant in *B3GLCT* (c. 660 + 1 G > A splice donor; Invitae, Pediatric Genetics Specialty Practice, 60 W Gore St, Orlando, FL 32806, Oct 2017). His axial length measurements and B scan ultrasound estimate of cupping were both stable compared to baseline. Patient was monitored on the same medications without surgical intervention. Genetic screening were offered to patient’s parents but were declined.

**Fig. 1:**
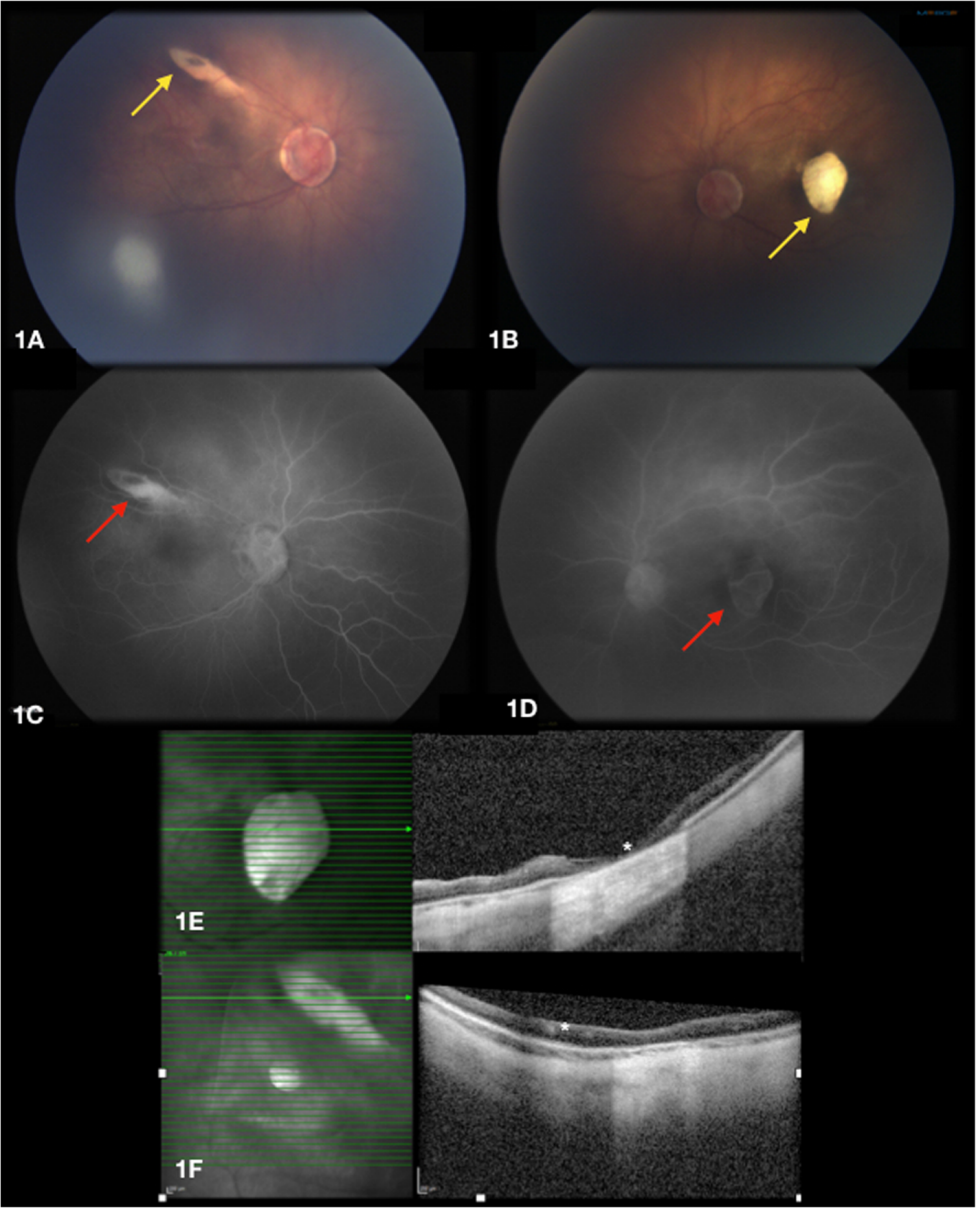
**a** (right eye) and **b** (left eye) show the color fundus photos, areas of demarcated severe chorioretinal atrophy can be seen (yellow arrows). **c** (right eye) and **d**(left eye) show late phases of fluorescein angiography with hyperfluorescence of the atrophic lesions (red arrows) 1E (right eye) and **f** (left eye) show scan of spectrum domain optic coherence tomography trough the areas of atrophy, generalized thinning of all retina layers and choroid are demonstrated (white asterisks)

In the subsequent serial EUA, more adhesions between the iris and cornea OS were noted despite the generally improved corneal opacities OU. Posterior segment improved as the media opacity cleared. Fluorescein angiography (FA) was performed (RetCam3, Ophthalmic Imaging System, Natus Medical), and spectral-domain optical coherence tomography (SD-OCT) images were obtain (Spectralis flex module, Heidelberg Engineering). The optic discs were noted to have morning glory-like appearances suggestive of colobomatous changes, with a cup to disc ratio of approximately 0.7 OU. Diffuse chorioretinal atrophies were noted [Fig. 1a-b]. The right eye had a well-defined white-yellowish, comet shaped lesion along the superotemporal arcade [Fig. 1 a]. A similar lesion that was well-circumscribed, oval-shaped and atrophic was seen in the central macula of the left eye [Fig. 1 b]. FA demonstrated early hyperfluorescence of the lesion with no leakage in the right eye, and early hyperfluorescence secondary to a window defect corresponding to the macular lesion in the left eye. Late frames of the FA showed hyperfluorescence of the atrophic lesions [Fig. 1 c-d]. There were neither leakage from the optic disc, nor vascular abnormalities in the peripheral retina. Intraoperative SD-OCT of the macula showed severe diffuse thinning of the retina and choroid in the atrophic lesions. Additionally, in the left eye oval shaped atrophic lesion, a complete loss of retinal structure was noted [Fig. 1E-F]. Written informed consent to publish the acquired images were obtained from the patient’s guardians.

## Discussion and conclusion

In this case report, we used multimodal imaging to characterize the bilateral chorioretinal lesions in a young patient with PPS associated with a homozygous *B3GLCT* mutation. Both eyes had diffuse chorioretinal atrophy, with very well-defined areas of thinning/absence of the retina layers located in the posterior pole. Reis et al. described a retinal coloboma-like lesion in a premature PPS patient with *B3GLCT* mutation [6]. However, the lesion was not further characterized in their report [6]. Weh and colleagues reported one patient with chorioretinal changes in their cohort of 9 PPS patients with *B3GLCT* mutations, but a detailed description of the lesion was similarly not presented [7].

The *B3GLCT* gene (chromosome 13) was identified by Heinonen et al. and encodes a glucosyltransferase protein which catalyzes glycosylation of other proteins [8]. This protein mediates the non-canonical endoplasmic reticulum quality-control mechanism, which involves identifying and stabilizing properly folded proteins through glycosylation [9]. As of 2019, all 15 *B3GLCT* mutations reported in the literature (including c.660 + 1G > A, the most common allele) were predicted to cause truncated protein products that result in complete loss of function [6, 10]. The *B3GLCT* gene transcripts have been found in various human tissues including the heart, kidney, and brain [8, 11]. A loss of function mutation in *B3GLCT* gene likely causes malfunctioning in protein glycosylation resulting in anomalies of multiple organ systems [8, 11]. The mechanism resulting in the chorioretinal findings remain uncertain.

There are several limitations to our case report. It is possible that the chorioretinal findings were either isolated events independent of the genetic mutation or were associated with other genetic defects that were not identified. The timing of the development of such chorioretinal changes was unclear in our patient. His corneal opacity initially precluded adequate examination of his posterior pole, potentially delaying the identification of the retinal abnormalities. Alternatively, the chorioretinal findings could be an acquired, progressive process rather than a congenital one. Despite these limitations, we believe our findings contribute to the phenotypic characterization of *B3GLCT* mutation and PPS.

## Data Availability

Data sharing is not applicable to this article as no datasets were generated or analyzed during the current study.

## Abbreviations

PPS: Peters plus syndrome
EUA: Exam under anesthesia
IOP: Intraocular pressures
OD: Right eye
OS: Left eye
OU: Both eyes
FA: Fluorescein angiography
SD-OCT: Spectral domain optical coherence tomography
